# Assembly of Recombinant Israeli Acute Paralysis Virus Capsids

**DOI:** 10.1371/journal.pone.0105943

**Published:** 2014-08-25

**Authors:** Junyuan Ren, Abigail Cone, Rebecca Willmot, Ian M. Jones

**Affiliations:** School of Biological Sciences, University of Reading, Reading, United Kingdom; University of British Columbia, Canada

## Abstract

The dicistrovirus Israeli Acute Paralysis Virus (IAPV) has been implicated in the worldwide decline of honey bees. Studies of IAPV and many other bee viruses in pure culture are restricted by available isolates and permissive cell culture. Here we show that coupling the IAPV major structural precursor protein ORF2 to its cognate 3C-like processing enzyme results in processing of the precursor to the individual structural proteins in a number of insect cell lines following expression by a recombinant baculovirus. The efficiency of expression is influenced by the level of IAPV 3C protein and moderation of its activity is required for optimal expression. The mature IAPV structural proteins assembled into empty capsids that migrated as particles on sucrose velocity gradients and showed typical dicistrovirus like morphology when examined by electron microscopy. Monoclonal antibodies raised to recombinant capsids were configured into a diagnostic test specific for the presence of IAPV. Recombinant capsids for each of the many bee viruses within the picornavirus family may provide virus specific reagents for the on-going investigation of the causes of honeybee loss.

## Introduction

An annual loss of honey bee colonies has been reported across the United States and more recently Europe, a proportion of which has been termed Colony Collapse Disorder (CCD) [1]. The consequential loss to agriculture as a result of reduced bee enabled pollination has been widely reported [2]. Honey bee loss and CCD may have multifactorial origins including loss of rural habitat, pesticide use, mite infestation and pathogen load [3]–[5]. Pathogenic bee viruses may play a role but as a number of common viruses are found, Kakugo virus; Varroa Destructor Virus; Sacbrood Virus; Deformed Wing Virus, Kashmir Bee Virus and Israeli Acute Paralysis Virus [6]–[8], a definitive link between any one infection and CCD has been difficult to establish. An early metagenomic analysis of hives that suffered CCD identified Israel Acute Paralysis Virus (IAPV) as a highly correlated risk factor [9] although other viruses, notably Deformed Wing Virus, have since been implicated. Infection is linked to Varroa mite infestation [10], [11]. While the precise causes of CCD remain unknown the horizontal transfer of pathogenic viruses from mites to bees is a possible factor in CCD with parallels in other cases of viruses crossing the species barrier [12], [13]. Direct evidence from areas of emerging CCD occurrence [14] or from model studies of Varroa stimulated virus transmission [15], [16] lends support to such a possibility. IAPV can be vectored by Varroa [17] and has recently been shown to concentrate in the heads of bees and alter foraging behaviour, plausibly leading to bee disorientation and loss [18].

Many of the known bee viruses belong to the *Picornaviridae* and *Dicistroviridae* families, both of which are characterised by icosahedral particles of a pseudo T = 3 symmetry and have similar protein coding and virus assembly pathways. In the environment multiply infected hives are common [19]–[21] which, with virus strain variation and limited permissive honey bee cell culture [22], [23], make studies on the pathogenic contribution attributable to any single infectious virus difficult.

In contrast, the assembly of non-infectious picornavirus empty capsids has been reported for several picornaviruses [24]–[27] and we showed recently that the level of such capsids could be improved by regulating the level of the 3C protease required for capsid protein maturation [28], [29]. Here we apply the same expression strategy to IAPV to show that empty IAPV capsids can be assembled in insect cells following expression using recombinant baculoviruses. Capsids allowed the generation of monoclonal antibodies (MAbs) whose epitopes were subsequently mapped. Two non-competing MAbs were configured as a capture ELISA assay that detected IAPV antigen in expressing cells. We suggest that recombinant empty capsid synthesis may be a generally useful tool for the generation of specific reagents for fundamental and applied studies of many honeybee viruses.

## Results

**Figure 1:**
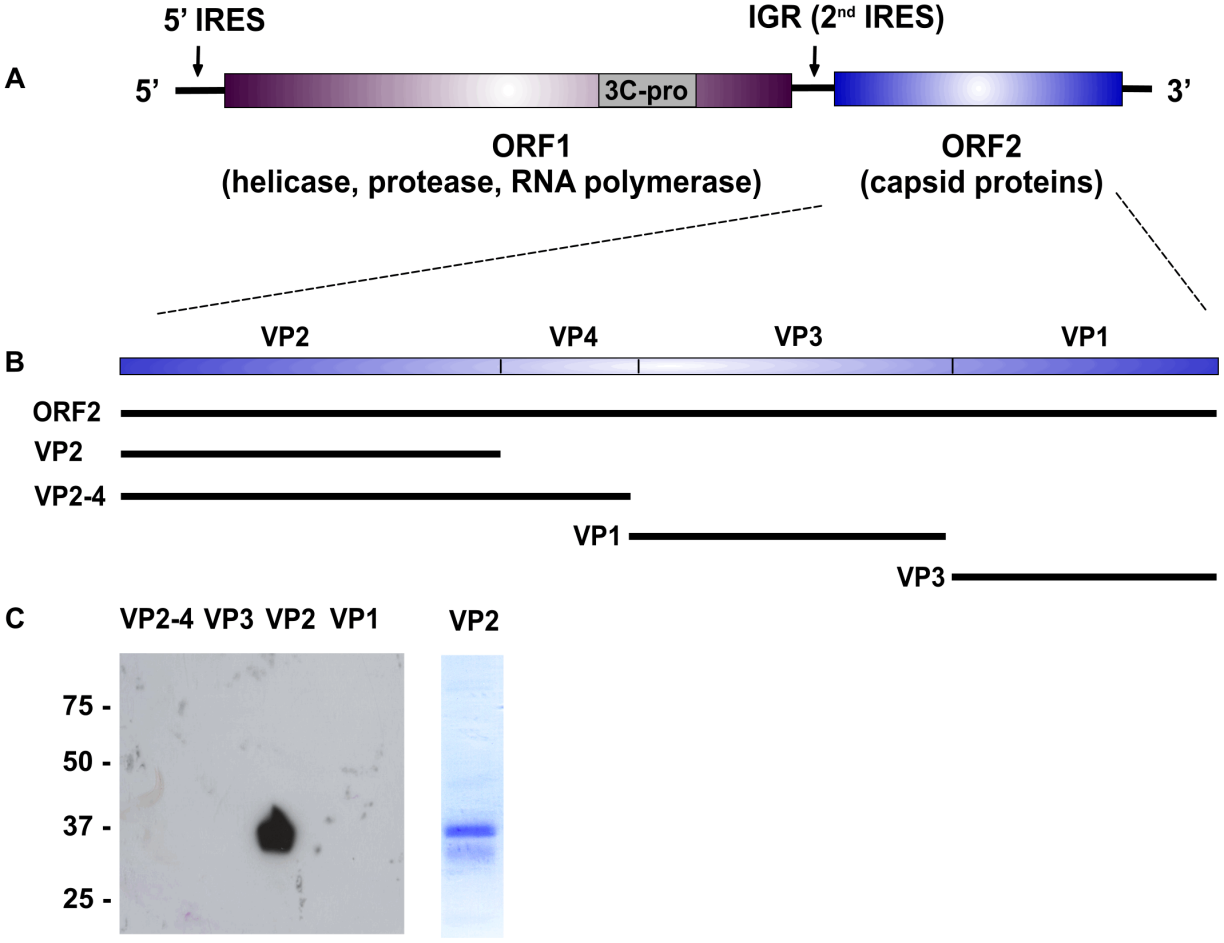
The IAPV genome and origin of sequences used for IAPV fragment expression. **A**. Genome structure of IAPV dicistrovirus with the two open reading frames and their constituent mature proteins shown. IRES – internal ribosome entry site, IGR – intergenic region. **B**. The fragments targeted for expression in *E.coli* and their encoded products. The precise endpoints used were: VP2, VTH→MQC; VP2-4, VTH→FGW; VP3, SKP→ELQ; VP1, INI→ISR. Expression screening for the proteins predicted from the fragments shown in B. **C**- left. Western blot with anti-His antibody. **C**-right. Purified VP2 used for the generation of a rabbit serum. Numbers to the left of the blot are protein molecular mass markers and are in kilodaltons.

First reported in 2007 [30], IAPV has since been found worldwide with at least 2 strains co-circulating in the United States and Canada [31]. IAPV has been independently isolated in France in a screen of apiary overwintering losses [32] and more recently in Argentina [33], Japan [34] China [20] and Spain [21], [35]. Despite this, there are relatively few specific regents for the non-nucleic acid based detection of IAPV particles. In order to allow detection of IAPV expression in a heterologous expression system we first generated a specific antibody for IAPV following a high throughput screen for stable expression of the predicted individual mature structural proteins of IAPV as His-tagged proteins in *E.coli* [36]. A synthetic IAPV ORF2 (NC_009025, codon optimised for *Spodoptera* cells) was used as template for the amplification of fragments encoding VP2+VP4, VP2, VP3 and VP1 with junctions based on an alignment of IAPV ORF2 with the known endpoints in *Solenopsis invicta* virus 1 where they have been formally mapped [37] (Figure 1A and 1B). Of the fragments screened, only that encoding VP2 gave rise to detectable amounts of protein (Figure 1C). Recombinant VP2 was purified by immobilised metal chromatography (Figure 1C) and the purified protein used to generate a high titre polyvalent serum in rabbits which did not cross react with insect cells (not shown).

**Figure 2:**
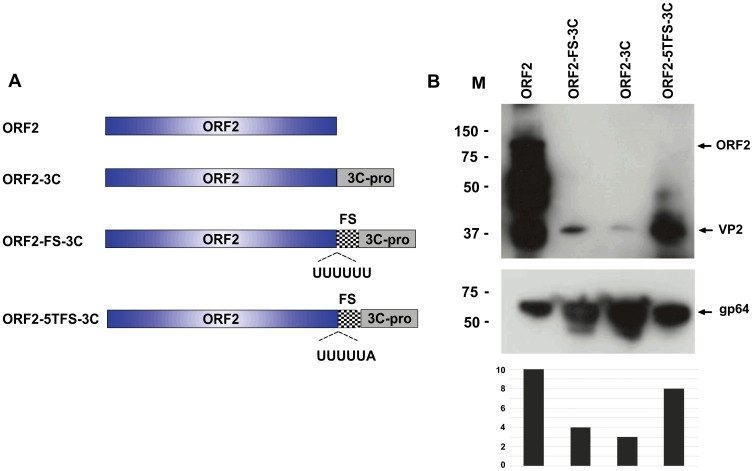
Design and test of baculovirus expression cassettes. **A**. Cartoon representations of the various genetic constructs used to assess IAPV antigen expression in infected Sf9 cells. The sequences used are identified. FS- the HIV-1 frameshift site, the polypyrimidine tract of which is indicated. 3C-pro – the IAPV 3C like protease. **B**. Western blots of Sf9 cells infected for 2 days with recombinant baculoviruses constructed with the cassettes shown in A. In B the upper panel was blotted with rabbit anti-VP2 serum, the middle panel with anti-gp64 and the lower panel is the relative VP2:gp64 level. Numbers to the left of the blots are protein molecular mass markers (M) and are in kilodaltons. The expected position of the blotted antigens is indicated.

To assess expression of IAPV ORF2 as an intact polyprotein and following cleavage into mature virus structural proteins we assembled baculovirus transfer vectors with the sequence encoding ORF2 as a sole open reading frame and, in other variants appended at the ORF2 carboxyl–terminus to the sequence encoding the putative IAPV 3C like protease responsible for ORF2 maturation, normally part of the ORF1 polyprotein. The 3C protease activity in many picornaviruses is associated with host cell shutdown through the cleavage of cellular targets e.g. [38] so we attenuated the level of 3C expression by the introduction of a ribosomal frameshift sequence between the sequence encoding ORF2 and that encoding IAPV 3C as described recently for Foot and Mouth Disease Virus [28]. In an additional modification we reduced the frameshift rate further through shortening of the “slippery sequence” where ribosomal slippage occurs from six to five uracils which reduces the frequency of the -1 frameshifting event [39], [40]. These constructions (Figure 2A) are predicted to reduce the level of 3C enzyme while maintaining the same level of ORF2 translation. Following sequence verification recombinant baculoviruses were produced as described [41] and high titre stocks were used to infect *Spodoptera frugiperda* (Sf9) cells. Infected cells were harvested at two days post infection and total cell extracts assessed by SDS-PAGE and western blot for the presence of VP2 related antigen, by probing with the generated rabbit anti-VP2 serum, and for level of infection by probing for the baculovirus major surface glycoprotein gp64. Expression of ORF2 alone resulted in very intense staining of a band at ∼100 kDa with a considerable number of smaller products, the result of non-specific proteolysis within the expressing Sf9 cells (Figure 2B track 1). When ORF2 was coupled in frame to the 3C protease the band at ∼100 kDa was wholly processed to a single species of ∼39 kDa consistent with the expected molecular weight of the mature VP2 product (Figure 2B track 3). However the level of signal was lower than ORF2 alone. When the 3C protease was coupled to the C terminus of ORF2 in the −1 frame via a ribosomal frameshift site precursor processing to VP2 was improved when compared to that observed following direct fusion (Figure 2B track 2). When the ribosomal frameshift frequency was reduced by use of a 5 U slippage sequence the level of the VP2 signal approached that of the unprocessed precursor (Figure 2B track 4). The level of baculovirus infection by these viruses was broadly similar as defined by the level of the baculovirus encoded gp64 protein (Figure 2B middle panel). Thus IAPV 3C processes the ORF2 precursor following expression in Sf9 cells and the level of cleavage observed is affected by the ratio of enzyme to substrate (Figure 2B lower panel).

**Figure 3:**
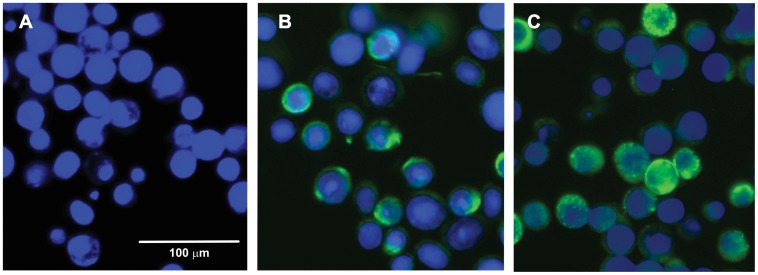
Immunofluorescence of Sf9 cells infected for 2 days with constructs ORF2 and ORF2-5TFS-3C stained with anti IAPV VP2 and an anti-rabbit Alexa Fluor 488 conjugate. **A** – Sf9 mock infected control. **B** – Sf9 cells infected with construct ORF2 expressing ORF2 only. **C** – Sf9 cells infected with construct ORF2-5TFS-3C expressing ORF2 fused with an attenuated 3C like protease. Occasional punctate staining is apparent in C.

**Figure 4:**
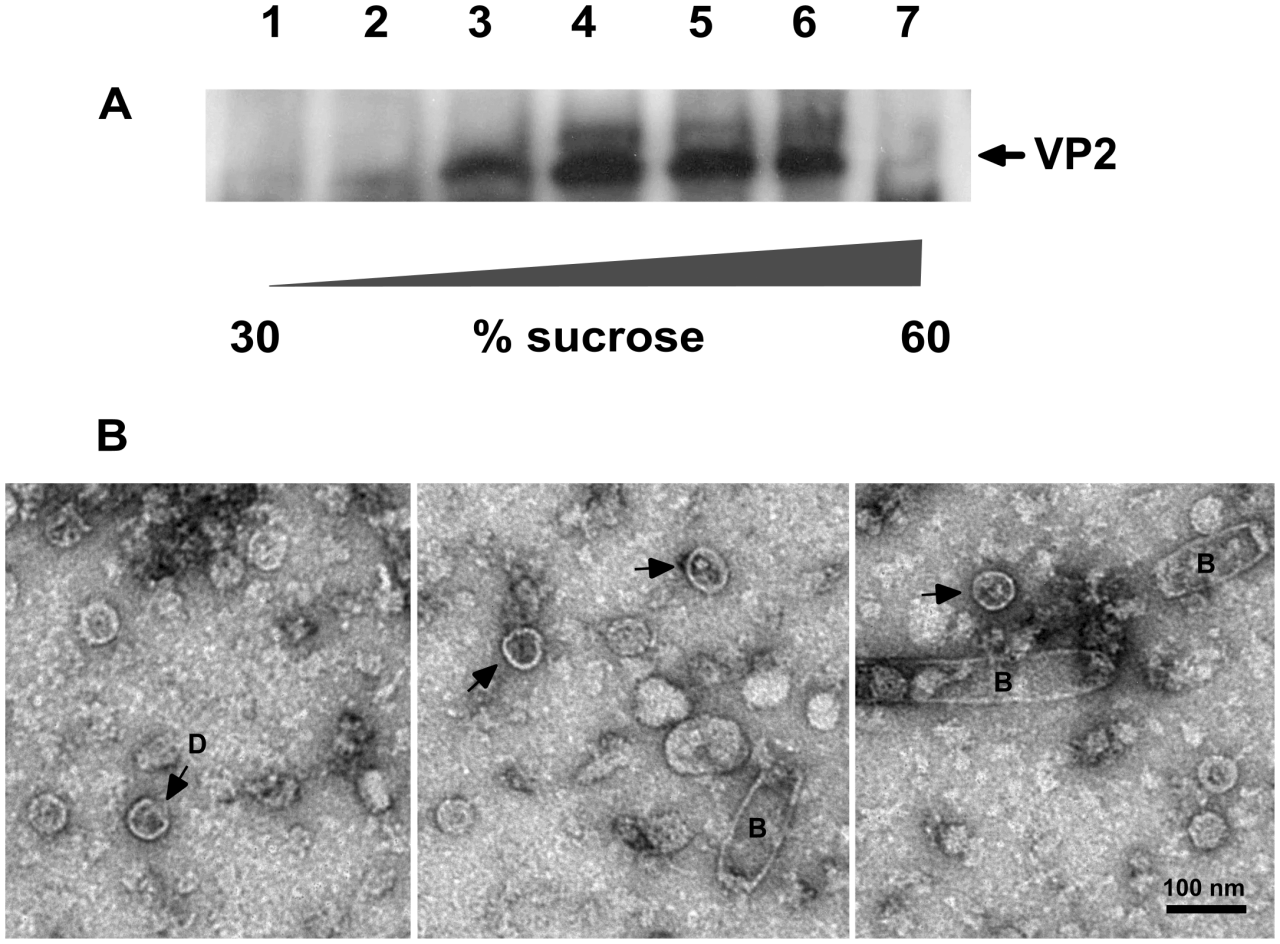
Analysis of empty IAPV capsids. **A**. Sucrose gradient analysis of Sf9 cell extracts infected with recombinant baculovirus ORF2-5TFS-3C. Each fraction represents a 5% step from 30% to 60% sucrose w/v and is blotted with the IAPV anti VP2 serum. **B**. EM analysis of peak fractions from the sucrose gradient shown in A. Empty capsids are indicated. A damaged particle is labelled D. Baculovirus nucleocapsids are labelled B. The bar is 100 nm.

To confirm IAPV protein expression as a result of Sf9 cell infection by recombinant baculoviruses, infected cells at two days post infection were fixed and permeablised and incubated with anti VP2 serum followed by a fluorescent anti-rabbit conjugate and visualised by fluorescence microscopy. Expression of ORF2 alone gave rise to bright fluorescence distributed throughout the cytoplasm (Figure 3A). The signal from the optimised (frameshift with 5Ts) recombinant, ORF2-5T-3C, was somewhat reduced but also distributed within the expressing cell although occasional punctate staining was apparent (Figure 3B). These data suggest that processing of the IAPV structural precursor protein may be associated with some redistribution of recombinant antigen within the expressing cell, plausibly as a result of assembly of the cleaved structural proteins into discistrovirus like capsids. To confirm that capsid assembly was occurring in this heterologous expression system, cells infected with the optimised recombinant baculovirus expressing ORF2-5T-3C were lysed at two days post infection and the cytoplasmic contents fractionated on sucrose velocity gradients. VP2 reactive antigen was found in the middle fractions, typically around 40% sucrose, reflecting a higher molecular weight than would be expected for VP2 alone (Figure 4A). When peak fractions from the gradient were absorbed onto carbon coated formvar grids and analysed by transmission electron microscopy, particles with the shape and dimensions typical of assembled dicistrovirus capsids were observed in addition to baculovirus nucleocapsids which co-sedimented in the same sucrose fractions (Figure 4B). Many recombinant dicistrovirus capsids had taken up stain showing them to be hollow as expected of assembly in the absence of a packageable genome (Figure 4B arrowed). Control extracts processed from baculovirus only infected cells showed no such particles, only baculovirus nucleocapsids (not shown). Some particles appeared damaged and other material of regular size would be consistent with disassembled capsids. A high level of disassembled capsid material has also been observed in EM grids of Triatoma virus [42]. Well defined particles did not show the obvious angular profile typical of picornavirus icosahedra reflecting the fact that dicistrovirus structures, while possessing icosahedral symmetry, are generally more spherical, shown classically for cricket paralysis virus [43] and more recently for Infectious flacherie virus [44] although projections around the 5 fold axes were observed in the recently solved Triatoma dicistrovirus structure [45].

**Figure 5:**
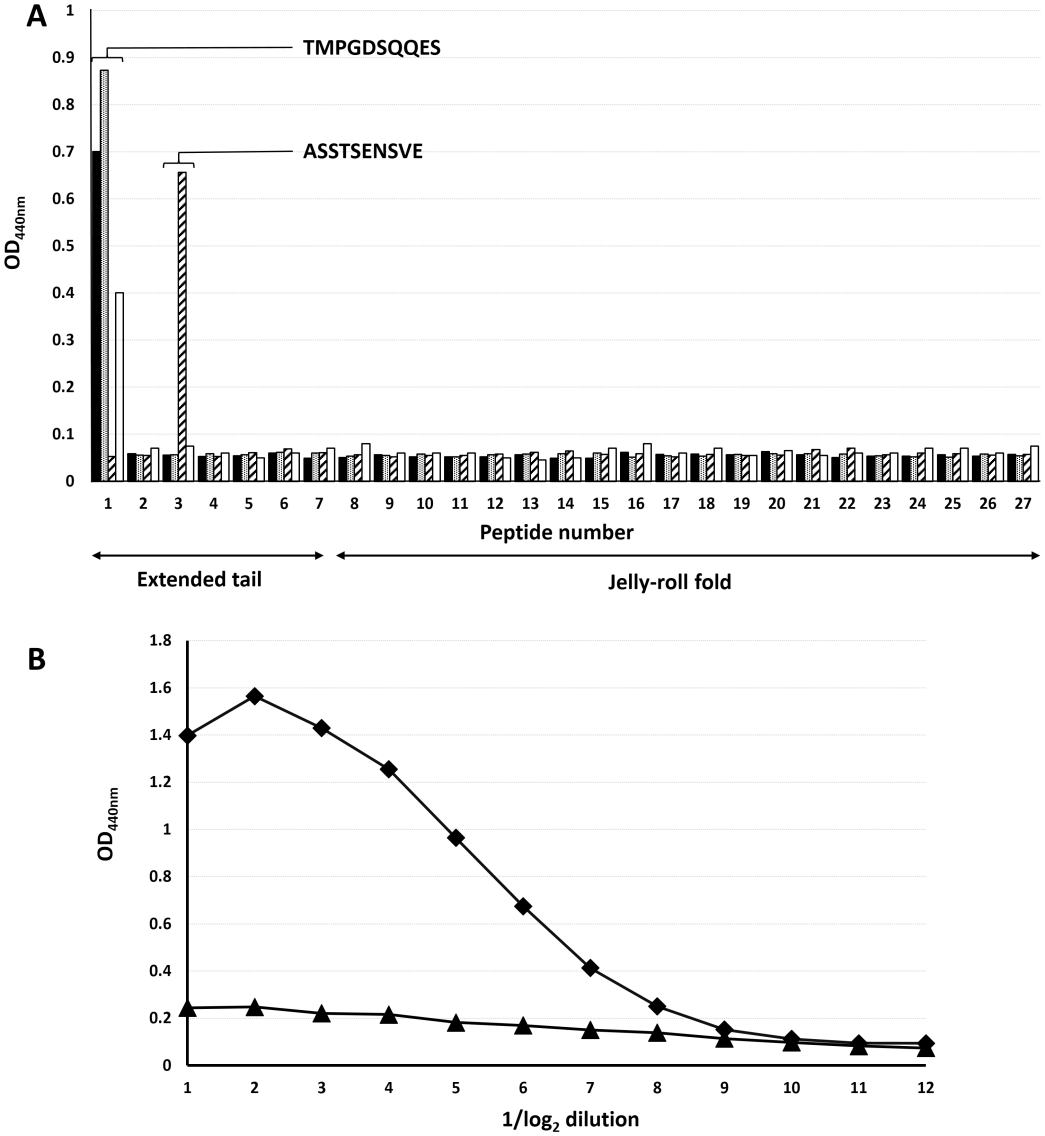
Development of an IAPV capture ELISA. **A**. Pepscan of 4 monoclonal antibodies to IAPV VP2 on an overlapping peptide library. Filled bars IAPVMAb8; stipple bars IAPVMAb12; striped bars IAPVMAb17; open bars IAPVMAb 27. The relationship between peptide identity and VP2 structure is shown. **B**. Twin site capture ELISA using capture MAbs 8 (triangles) and 27 (diamonds) as capture layer and probing with HRP-labelled IAPV MAb12. The test sample was a lysate of Sf9 cell infected with the baculovirus expressing ORF-2-5TFS-3C.

The predominant diagnostic test for IAPV presence is nucleic acid amplification using specific primers e.g. [46], a very sensitive but technologically demanding assay. To enable the development of an antibody based diagnostic assay as an alternative tool that could be used in field conditions, we generated monoclonal antibodies to part purified recombinant IAPV empty capsids, screening hybridomas in the first instance on purified VP2 protein. Four strongly reactive antibodies, IAPVMAb8, IAPVMAb12, IAPVMAb17 and IAPVMAb27 were selected for formal epitope mapping using a library of 20 residue peptides overlapping by 10 made to the same IAPV VP2 sequence. Interestingly all four antibodies reacted with the N-terminal extended tail of VP2 visualised in the available dicistrovirus structures [43], [45]. Three antibodies co-mapped to the extreme N-terminal sequence TMPGDSQQES and one to an adjacent sequence ASSTSENSVE (Figure 5A). As predicted by the mapping, IAPVMAbs 8, 12 and 17 competed when used as pairs in a twin site ELISA assay with ORF2-5T-3C infected Sf9 cell extracts as the test antigen but a combination of IAPVMAbs 12 and 27 were found to allow a dose dependent detection of IAPV antigen in the test lysates (Figure 5B). The antibody pairs had no reaction to Sf9 cells that were not infected with an IAPV antigen expressing baculovirus or bee lysate (not shown).

## Discussion

We have assembled empty IAPV capsids in insect cells following expression of IAPV structural proteins using a recombinant baculovirus. The mature structural proteins were produced by cleavage of a precursor protein, ORF2, by the authentic 3C-like processing enzyme which was relocated from ORF1 and expressed as a C-terminal fusion protein. Direct fusion of 3C to ORF2 showed only low levels of cleaved VP2 whereas lowering the expression level of 3C through a translational control strategy resulted in higher levels of mature capsid expression. This is consistent with IAPV 3C activity exhibiting some cytotoxicity as has been described for picornavirus 3C where moderation of 3C activity through a mixture of translational control and 3C mutation resulted in higher empty capsid yields [28]. In the case of IAPV, a structure of the 3C-like enzyme is not available so translational control through the use of a frameshift sequence known to function in insect cells [47] was the sole option. It is likely a similar strategy will work for other members of the *Picornaviridae* or *Dicistroviridae* where reduced 3C activity is required to maximise capsid cleavage. Recombinant empty IAPV particles with typical dicistrovirus appearance were observed by EM of negatively stained sucrose gradient fractions and were immunogenic upon immunization. Monoclonal antibodies selected to VP2 were found to map exclusively to the extended N-terminal tail of the molecule. Mapping the polyvalent response also showed this region to be immunodominant, consistent with a poorly immunogenic well folded jelly-roll capsid structure (not shown). Notwithstanding a restricted repertoire in the immune response, VP2 specific monoclonal antibody pairs were validated for ELISA detection of IAPV antigen expressed in *Spodoptera* cells. The two epitopes recognised are IAPV specific which suggests that a rapid test for IAPV presence, such as a lateral flow device, would be highly specific and suitable for use in the field. Such devices have been described for diagnostic tests for other bee pathogens and viruses [48], [49]. The number of viruses infecting agriculturally important insects such as the honeybee continues to grow and mixed infections are common [7], [10], [11], [15], [50]. Methods to provide the equivalent of a pure virus culture in the absence of the requirements for infectious plaque assay could be useful to generate highly specific protein based assays. Similarly, differences in individual variants of one virus, such as the recently described Varroa transmitted virulent form of DWV [16] could be assessed for capsid function through such technology. Through such studies, baculovirus expression of empty capsids following the co-expression of the structural precursor and a suitably attenuated 3C-like enzyme could contribute to better understanding the honeybee-virus landscape at a molecular level.

## Materials and Methods

### Cell culture and virus growth

Sf9 cells (ATCC) were cultured in BioWhittaker Insect-Xpress supplemented with 2% FCS, 100units/ml penicillin, 100 µg/ml streptomycin and 2.5 µg/ml amphotericin B. Cells were grown at 28°C as monolayers or in suspension with agitation at 100 rpm. Baculoviruses were generally amplified in monolayer cultures but large scale infections for capsid isolation were done in suspension. Virus stocks were titred using plaque assay on Sf9 monolayers.

### Sequences and cloning

Sequences for IAPV were taken from the databases (NC_009025) and synthesised *de novo* to include codon optimisation for *Spodoptera* cells (Geneart). The transfer vector used for all expression was based on pTriEx1.1 (EMD Bioscience) and fragments encoding ORF2 and 3C were cloned downstream of the P10 promoter via unique restriction sites introduced during gene synthesis. Recombinant baculoviruses were constructed using recombination with AcMNPV bacmid KO1629 in insect cells as described [41]. Routine DNA procedures used standard protocols or, when kits were used, those recommended by the vendor. All vectors were confirmed by DNA sequencing prior to use for expression.

### Electrophoresis and Western blotting and Quantitation

Protein samples were separated on pre-cast 10% Tris.HCl SDS-polyacrylamide gels (BioRad) and transferred to Immobilon-P membranes (Millipore) using a semi-dry blotter. For 10 well gels each sample loaded represented 5×10^4^ cells. Following transfer, filters were blocked for one hour at room temperature using PBS containing 0.1% v/v Tween-20 (PBS-T), 5% w/v milk powder. Primary antibodies were used at a dilution of 1∶5000 in PBS-T, 5% w/v milk powder. Following several washes with PBS-T the membranes were incubated for 1 hour with the appropriate HRP-conjugates and the bound antibodies detected by BM chemiluminescence (Roche). Bands were imaged using a Syngene G:Box chemiluminescence imager and the image file processed for pixel density by Genetools software (Syngene).

### Purification of empty capsids

Infected insect cell cultures (typically 1 L at ∼10^6^/ml) were harvested at 3 days pi and lysed by resuspension in 1/20th of the original volume of 1% Triton X-100 in PBS and held at 4°C for 30 minutes with occasional agitation. Unbroken cells and nuclei were removed by centrifugation (4500 rpm, 30 min) and the clarified lysate layered onto a 30% sucrose (w/v in PBS) cushion and particles recovered by centrifugation at 100,000 g for 100 minutes. The gradient was discarded and the pellet resuspended in 1/10^th^ tube volume of PBS containing 3500 units of benzonase. After 30 min at room temperature the solution was clarified and applied to a preformed sucrose gradient made up of seven steps of 5% from 30% to 60% w/v. The gradient was developed by centrifugation at 100,000 g for 16 hrs and fractionated from the top.

### Electron microscopy

Samples were allowed to adhere to carbon coated formvar grids for 5 min at room temperature followed by a brief water wash (1 min) before staining with 1% uranyl acetate for 1 min. Excess stain was removed by blotting and the grids examined on a Philips CM20 operating at 80 kV.

### Monoclonal antibody production

Monoclonal antibodies were produced under subcontract at FERA, Sand Hutton, UK. Briefly, three mice were immunised with 0.1 ml of recombinant IAPV empty capsids at 300 µg/ml four times over an 8 week period. Following the last immunisation, animals were sacrificed and splenocytes fused with myeloma cells using polyethylene glycol 1500 as the fusion agent. Fusion cell products were cultured in multi-well plates and cell culture supernatants (CCS) harvested for screening on IAPV VP2 protein at 12 days post fusion. Cell lines producing strongly reactive antibody were cloned by limiting dilution and re-screened by ELISA to produce pure hybridomas. Following scale –up, the antibody in the CCS from selected cell lines was purified by Protein G chromatography, concentrated to ∼10 mgs/ml and stored at −80 degrees centigrade. Immunisation and work with vertebrate animals (mice) was performed under a UK Home Office project license in accordance with the UK Animals (Scientific Procedures) Act 1986 (UK Government 1986) using the principles outlined in the Home Office guidance ‘Antibody Production: Principles for Protocols of Minimal Severity’ (Home Office 2000). The work adhered to the principles of the National Centre for the Replacement, Refinement and Reduction of Animals in Research see http://www.nc3rs.org.uk/page.asp?id=871.

### Antibody labelling

To allow competition assays selected monoclonal antibodies were labelled with horse radish peroxidase using the Lightning-Link HRP Antibody Labeling Kit (Novus Biologicals).

### ELISA assay

Immulon II ELISA plates (Nunc) were coated with antigen or capture antibody at 10 µgs/ml in 200 mM sodium bicarbonate overnight at 4°C. Plates were blocked with 1% bovine serum albumin (BSA) in PBS containing 0.1% v/v Tween-20 (PBS-T) and then washed with PBS-T. Subsequent layers were incubated for 1 hour at 25°C and washed between layers with PBS-T. The plate was developed with 50 µl per well of tetramethylbenzidine (Europa Bioproducts UK) and the reaction stopped by the addition of 50 µl of 0.1 M H_2_SO_4_. Absorbance was measured at 440 nm using a GENios plate reader (Tecan UK). Experiments were performed in duplicate and the average plotted.

### Epitope mapping

Overlapping peptides of 20 residues overlapping by 10 residues to the VP2 sequence of IAPV and biotinylated at the N-terminus were supplied by Think Peptides (UK). Peptides were dissolved in DMSO at ∼10 mgs/ml and added to streptavidin coated plates at 10 µg/ml. After 1 hr incubation to allow peptide capture the plate was blocked and treated as described for ELISA.
